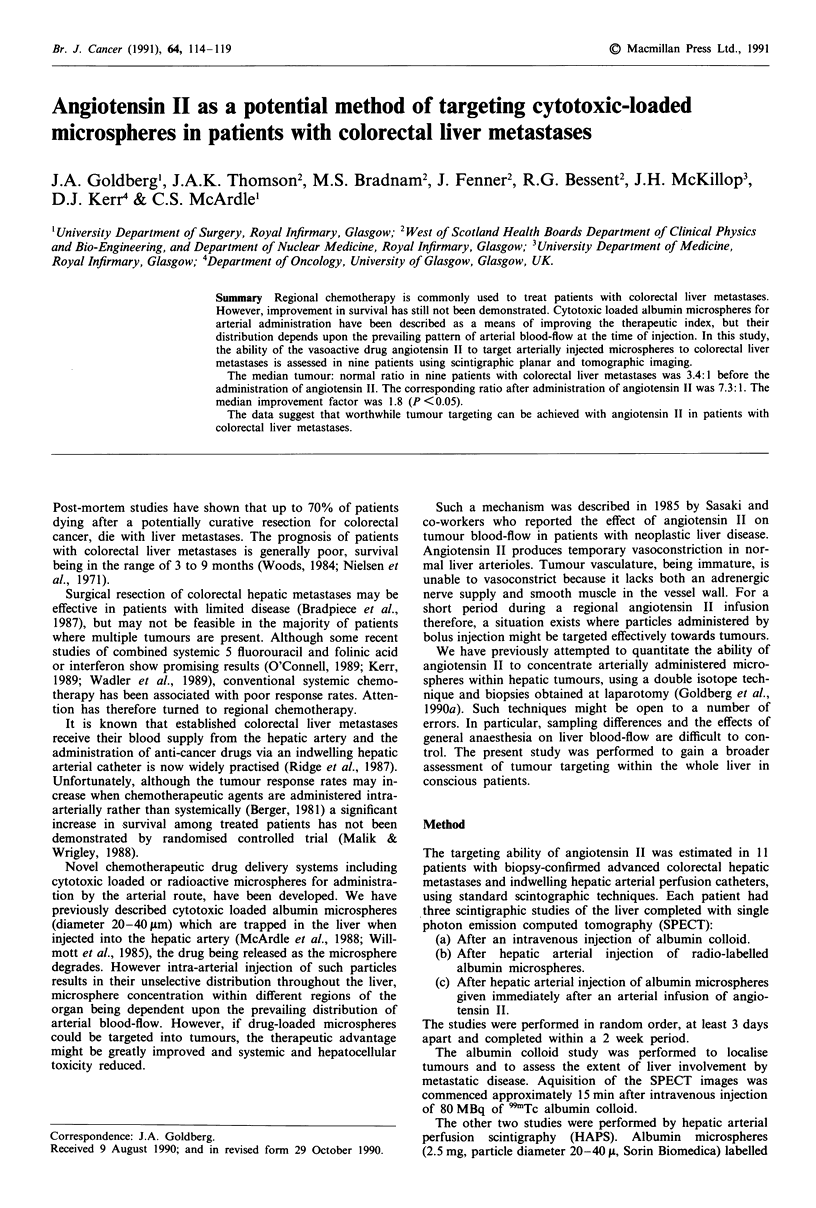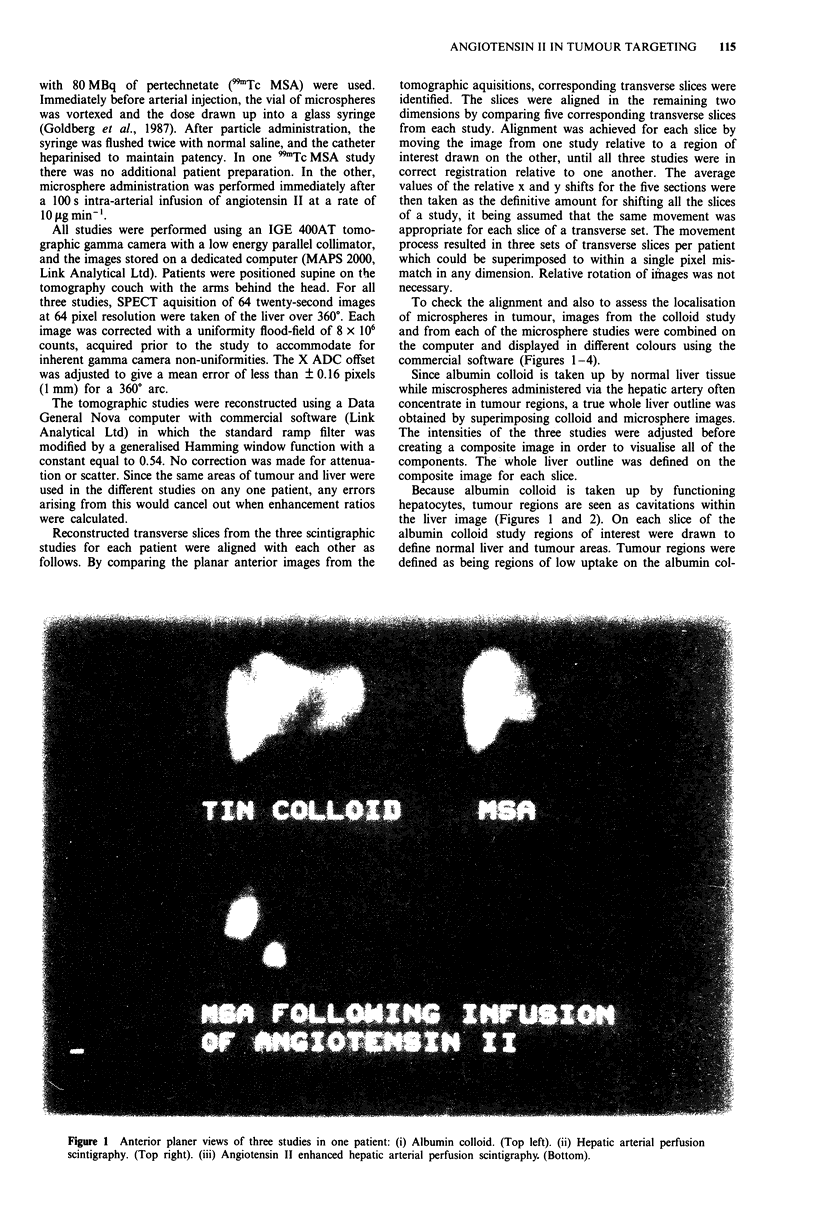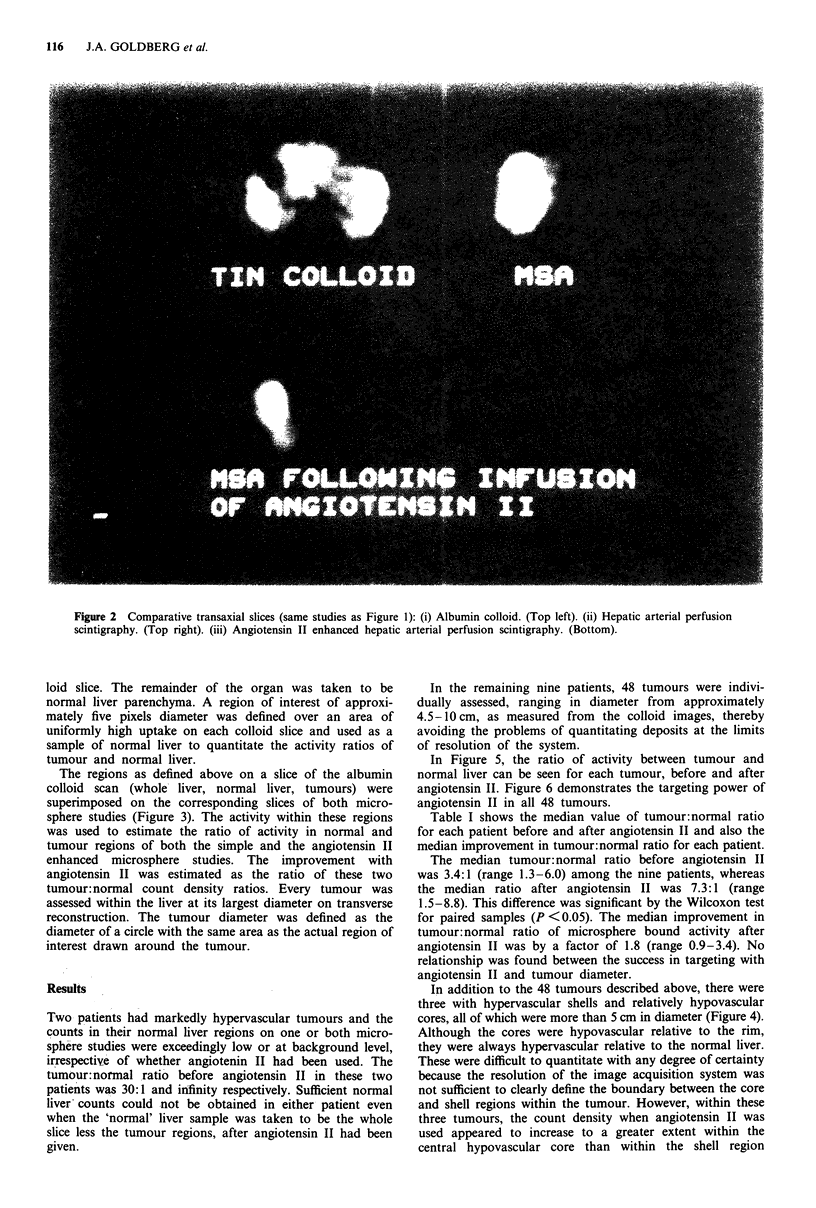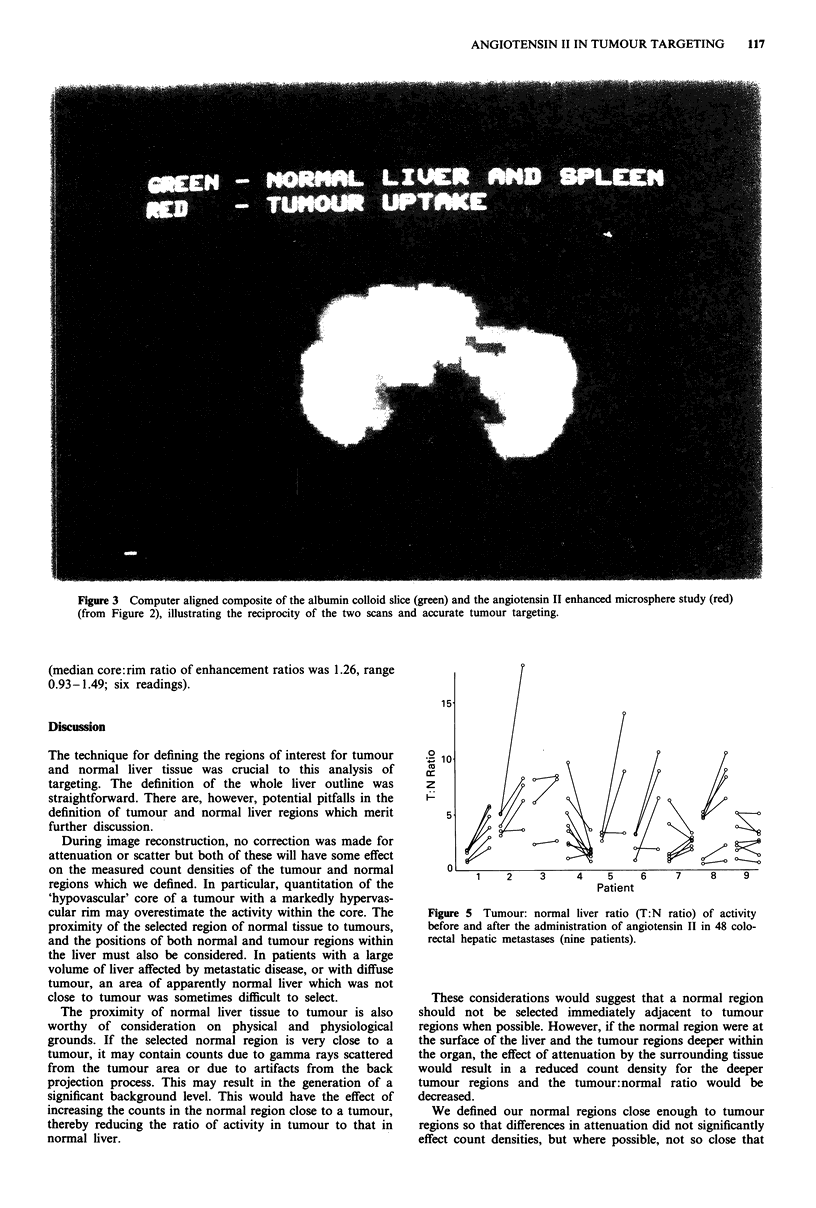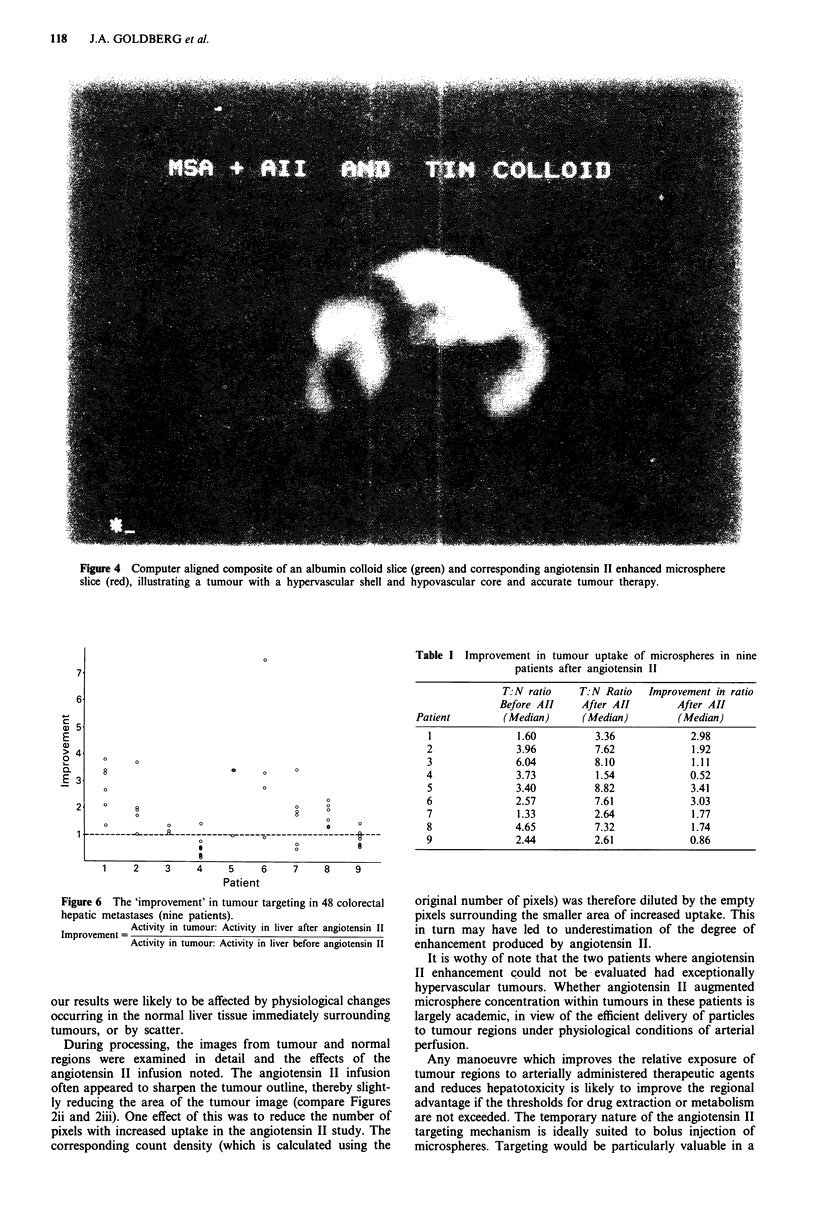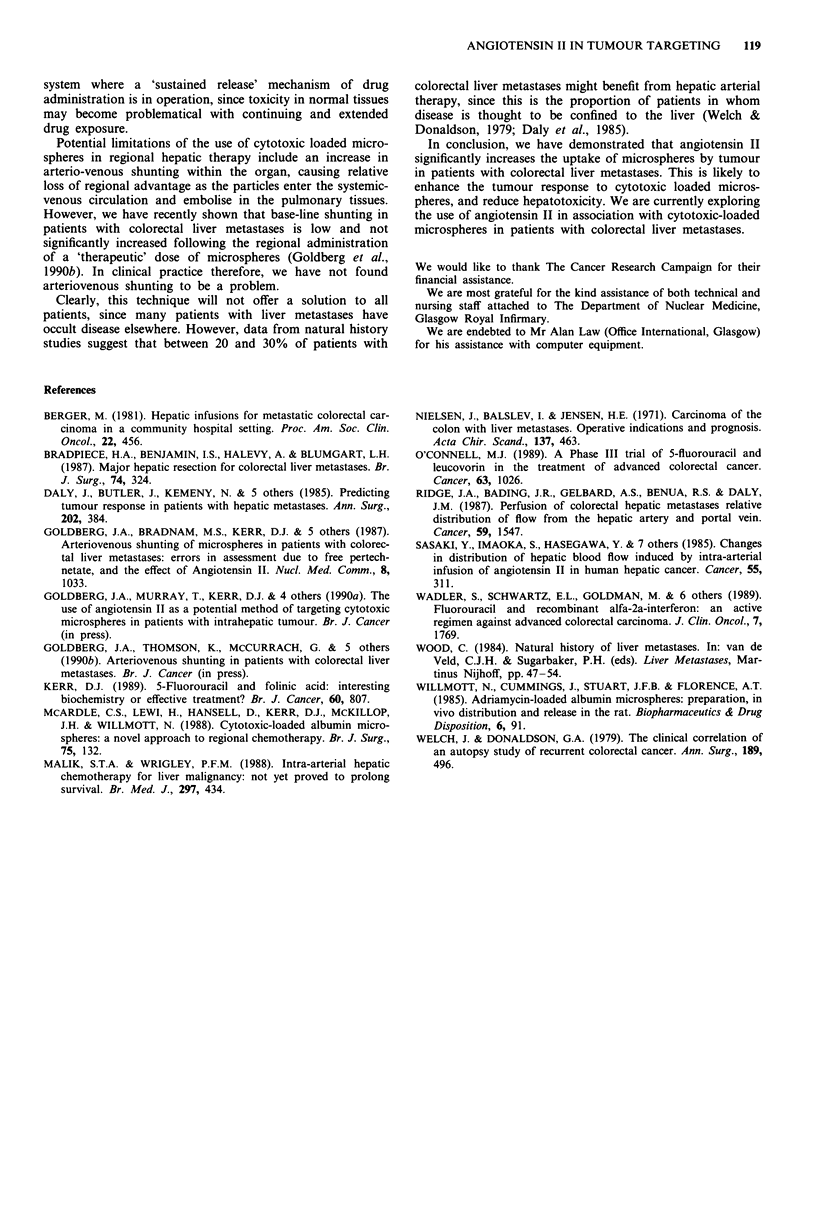# Angiotensin II as a potential method of targeting cytotoxic-loaded microspheres in patients with colorectal liver metastases.

**DOI:** 10.1038/bjc.1991.252

**Published:** 1991-07

**Authors:** J. A. Goldberg, J. A. Thomson, M. S. Bradnam, J. Fenner, R. G. Bessent, J. H. McKillop, D. J. Kerr, C. S. McArdle

**Affiliations:** University Department of Surgery, Royal Infirmary, Glasgow, UK.

## Abstract

**Images:**


 © Macmillan Press Ltd., 1991

### Angiotensin II as a potential method of targeting cytotoxic-loaded microspheres in patients with colorectal liver metastases

J.A. Goldberg[1], J.A.K. Thomson[2], M.S. Bradnam[2], J. Fenner[2], R.G. Bessent[2], J.H. McKillop[3], D.J. Kerr[4] & C.S. McArdle[1]

[1]*University Department of Surgery, Royal Infirmary, Glasgow;* [2]*West of Scotland Health Boards Department of Clinical Physics and Bio-Engineering, and Department of Nuclear Medicine, Royal Infirmary, Glasgow;* [3]*University Department of Medicine, Royal Infirmary, Glasgow;* [4]*Department of Oncology, University of Glasgow, Glasgow, UK.*

**Summary** Regional chemotherapy is commonly used to treat patients with colorectal liver metastases. However, improvement in survival has still not been demonstrated. Cytotoxic loaded albumin microspheres for arterial administration have been described as a means of improving the therapeutic index, but their distribution depends upon the prevailing pattern of arterial blood-flow at the time of injection. In this study, the ability of the vasoactive drug angiotensin II to target arterially injected microspheres to colorectal liver metastases is assessed in nine patients using scintigraphic planar and tomographic imaging.

The median tumour: normal ratio in nine patients with colorectal liver metastases was 3.4:1 before the administration of angiotensin II. The corresponding ratio after administration of angiotensin II was 7.3:1. The median improvement factor was 1.8 ($P < 0.05$).

The data suggest that worthwhile tumour targeting can be achieved with angiotensin II in patients with colorectal liver metastases.

Post-mortem studies have shown that up to 70% of patients dying after a potentially curative resection for colorectal cancer, die with liver metastases. The prognosis of patients with colorectal liver metastases is generally poor, survival being in the range of 3 to 9 months (Woods, 1984; Nielsen *et al.*, 1971).

Surgical resection of colorectal hepatic metastases may be effective in patients with limited disease (Bradpiece *et al.*, 1987), but may not be feasible in the majority of patients where multiple tumours are present. Although some recent studies of combined systemic 5 fluorouracil and folinic acid or interferon show promising results (O'Connell, 1989; Kerr, 1989; Wadler *et al.*, 1989), conventional systemic chemotherapy has been associated with poor response rates. Attention has therefore turned to regional chemotherapy.

It is known that established colorectal liver metastases receive their blood supply from the hepatic artery and the administration of anti-cancer drugs via an indwelling hepatic arterial catheter is now widely practised (Ridge *et al.*, 1987). Unfortunately, although the tumour response rates may increase when chemotherapeutic agents are administered intra-arterially rather than systemically (Berger, 1981) a significant increase in survival among treated patients has not been demonstrated by randomised controlled trial (Malik & Wrigley, 1988).

Novel chemotherapeutic drug delivery systems including cytotoxic loaded or radioactive microspheres for administration by the arterial route, have been developed. We have previously described cytotoxic loaded albumin microspheres (diameter 20–40 μm) which are trapped in the liver when injected into the hepatic artery (McArdle *et al.*, 1988; Willmott *et al.*, 1985), the drug being released as the microsphere degrades. However intra-arterial injection of such particles results in their unselective distribution throughout the liver, microsphere concentration within different regions of the organ being dependent upon the prevailing distribution of arterial blood-flow. However, if drug-loaded microspheres could be targeted into tumours, the therapeutic advantage might be greatly improved and systemic and hepatocellular toxicity reduced.

Such a mechanism was described in 1985 by Sasaki and co-workers who reported the effect of angiotensin II on tumour blood-flow in patients with neoplastic liver disease. Angiotensin II produces temporary vasoconstriction in normal liver arterioles. Tumour vasculature, being immature, is unable to vasoconstrict because it lacks both an adrenergic nerve supply and smooth muscle in the vessel wall. For a short period during a regional angiotensin II infusion therefore, a situation exists where particles administered by bolus injection might be targeted effectively towards tumours.

We have previously attempted to quantitate the ability of angiotensin II to concentrate arterially administered microspheres within hepatic tumours, using a double isotope technique and biopsies obtained at laparotomy (Goldberg *et al.*, 1990*a*). Such techniques might be open to a number of errors. In particular, sampling differences and the effects of general anaesthesia on liver blood-flow are difficult to control. The present study was performed to gain a broader assessment of tumour targeting within the whole liver in conscious patients.

### Method

The targeting ability of angiotensin II was estimated in 11 patients with biopsy-confirmed advanced colorectal hepatic metastases and indwelling hepatic arterial perfusion catheters, using standard scintographic techniques. Each patient had three scintigraphic studies of the liver completed with single photon emission computed tomography (SPECT):

(a) After an intravenous injection of albumin colloid.
(b) After hepatic arterial injection of radio-labelled albumin microspheres.
(c) After hepatic arterial injection of albumin microspheres given immediately after an arterial infusion of angiotensin II.

The studies were performed in random order, at least 3 days apart and completed within a 2 week period.

The albumin colloid study was performed to localise tumours and to assess the extent of liver involvement by metastatic disease. Aquisition of the SPECT images was commenced approximately 15 min after intravenous injection of 80 MBq of $^{99m}$Tc albumin colloid.

The other two studies were performed by hepatic arterial perfusion scintigraphy (HAPS). Albumin microspheres (2.5 mg, particle diameter 20–40 μ, Sorin Biomedica) labelled

Correspondence: J.A. Goldberg.
Received 9 August 1990; and in revised form 29 October 1990.

with 80 MBq of pertechnetate ($^{99m}$Tc MSA) were used. Immediately before arterial injection, the vial of microspheres was vortexed and the dose drawn up into a glass syringe (Goldberg *et al.*, 1987). After particle administration, the syringe was flushed twice with normal saline, and the catheter heparinised to maintain patency. In one $^{99m}$Tc MSA study there was no additional patient preparation. In the other, microsphere administration was performed immediately after a 100 s intra-arterial infusion of angiotensin II at a rate of 10 $\mu$g min$^{-1}$.

All studies were performed using an IGE 400AT tomographic gamma camera with a low energy parallel collimator, and the images stored on a dedicated computer (MAPS 2000, Link Analytical Ltd). Patients were positioned supine on the tomography couch with the arms behind the head. For all three studies, SPECT aquisition of 64 twenty-second images at 64 pixel resolution were taken of the liver over 360°. Each image was corrected with a uniformity flood-field of $8 \times 10^6$ counts, acquired prior to the study to accommodate for inherent gamma camera non-uniformities. The X ADC offset was adjusted to give a mean error of less than $\pm$ 0.16 pixels (1 mm) for a 360° arc.

The tomographic studies were reconstructed using a Data General Nova computer with commercial software (Link Analytical Ltd) in which the standard ramp filter was modified by a generalised Hamming window function with a constant equal to 0.54. No correction was made for attenuation or scatter. Since the same areas of tumour and liver were used in the different studies on any one patient, any errors arising from this would cancel out when enhancement ratios were calculated.

Reconstructed transverse slices from the three scintigraphic studies for each patient were aligned with each other as follows. By comparing the planar anterior images from the tomographic aquisitions, corresponding transverse slices were identified. The slices were aligned in the remaining two dimensions by comparing five corresponding transverse slices from each study. Alignment was achieved for each slice by moving the image from one study relative to a region of interest drawn on the other, until all three studies were in correct registration relative to one another. The average values of the relative x and y shifts for the five sections were then taken as the definitive amount for shifting all the slices of a study, it being assumed that the same movement was appropriate for each slice of a transverse set. The movement process resulted in three sets of transverse slices per patient which could be superimposed to within a single pixel mismatch in any dimension. Relative rotation of images was not necessary.

To check the alignment and also to assess the localisation of microspheres in tumour, images from the colloid study and from each of the microsphere studies were combined on the computer and displayed in different colours using the commercial software (Figures 1–4).

Since albumin colloid is taken up by normal liver tissue while miscrospheres administered via the hepatic artery often concentrate in tumour regions, a true whole liver outline was obtained by superimposing colloid and microsphere images. The intensities of the three studies were adjusted before creating a composite image in order to visualise all of the components. The whole liver outline was defined on the composite image for each slice.

Because albumin colloid is taken up by functioning hepatocytes, tumour regions are seen as cavitations within the liver image (Figures 1 and 2). On each slice of the albumin colloid study regions of interest were drawn to define normal liver and tumour areas. Tumour regions were defined as being regions of low uptake on the albumin col-

**Figure 1** Anterior planer views of three studies in one patient: (i) Albumin colloid. (Top left). (ii) Hepatic arterial perfusion scintigraphy. (Top right). (iii) Angiotensin II enhanced hepatic arterial perfusion scintigraphy. (Bottom).

**Figure 2** Comparative transaxial slices (same studies as Figure 1): (i) Albumin colloid. (Top left). (ii) Hepatic arterial perfusion scintigraphy. (Top right). (iii) Angiotensin II enhanced hepatic arterial perfusion scintigraphy. (Bottom).

loid slice. The remainder of the organ was taken to be normal liver parenchyma. A region of interest of approximately five pixels diameter was defined over an area of uniformly high uptake on each colloid slice and used as a sample of normal liver to quantitate the activity ratios of tumour and normal liver.

The regions as defined above on a slice of the albumin colloid scan (whole liver, normal liver, tumours) were superimposed on the corresponding slices of both microsphere studies (Figure 3). The activity within these regions was used to estimate the ratio of activity in normal and tumour regions of both the simple and the angiotensin II enhanced microsphere studies. The improvement with angiotensin II was estimated as the ratio of these two tumour:normal count density ratios. Every tumour was assessed within the liver at its largest diameter on transverse reconstruction. The tumour diameter was defined as the diameter of a circle with the same area as the actual region of interest drawn around the tumour.

### Results

Two patients had markedly hypervascular tumours and the counts in their normal liver regions on one or both microsphere studies were exceedingly low or at background level, irrespective of whether angiotenin II had been used. The tumour:normal ratio before angiotensin II in these two patients was 30:1 and infinity respectively. Sufficient normal liver counts could not be obtained in either patient even when the 'normal' liver sample was taken to be the whole slice less the tumour regions, after angiotensin II had been given.

In the remaining nine patients, 48 tumours were individually assessed, ranging in diameter from approximately 4.5–10 cm, as measured from the colloid images, thereby avoiding the problems of quantitating deposits at the limits of resolution of the system.

In Figure 5, the ratio of activity between tumour and normal liver can be seen for each tumour, before and after angiotensin II. Figure 6 demonstrates the targeting power of angiotensin II in all 48 tumours.

Table I shows the median value of tumour:normal ratio for each patient before and after angiotensin II and also the median improvement in tumour:normal ratio for each patient.

The median tumour:normal ratio before angiotensin II was 3.4:1 (range 1.3–6.0) among the nine patients, whereas the median ratio after angiotensin II was 7.3:1 (range 1.5–8.8). This difference was significant by the Wilcoxon test for paired samples ($P < 0.05$). The median improvement in tumour:normal ratio of microsphere bound activity after angiotensin II was by a factor of 1.8 (range 0.9–3.4). No relationship was found between the success in targeting with angiotensin II and tumour diameter.

In addition to the 48 tumours described above, there were three with hypervascular shells and relatively hypovascular cores, all of which were more than 5 cm in diameter (Figure 4). Although the cores were hypovascular relative to the rim, they were always hypervascular relative to the normal liver. These were difficult to quantitate with any degree of certainty because the resolution of the image acquisition system was not sufficient to clearly define the boundary between the core and shell regions within the tumour. However, within these three tumours, the count density when angiotensin II was used appeared to increase to a greater extent within the central hypovascular core than within the shell region

**Figure 3** Computer aligned composite of the albumin colloid slice (green) and the angiotensin II enhanced microsphere study (red) (from Figure 2), illustrating the reciprocity of the two scans and accurate tumour targeting.

(median core:rim ratio of enhancement ratios was 1.26, range 0.93–1.49; six readings).

### Discussion

The technique for defining the regions of interest for tumour and normal liver tissue was crucial to this analysis of targeting. The definition of the whole liver outline was straightforward. There are, however, potential pitfalls in the definition of tumour and normal liver regions which merit further discussion.

During image reconstruction, no correction was made for attenuation or scatter but both of these will have some effect on the measured count densities of the tumour and normal regions which we defined. In particular, quantitation of the 'hypovascular' core of a tumour with a markedly hypervascular rim may overestimate the activity within the core. The proximity of the selected region of normal tissue to tumours, and the positions of both normal and tumour regions within the liver must also be considered. In patients with a large volume of liver affected by metastatic disease, or with diffuse tumour, an area of apparently normal liver which was not close to tumour was sometimes difficult to select.

The proximity of normal liver tissue to tumour is also worthy of consideration on physical and physiological grounds. If the selected normal region is very close to a tumour, it may contain counts due to gamma rays scattered from the tumour area or due to artifacts from the back projection process. This may result in the generation of a significant background level. This would have the effect of increasing the counts in the normal region close to a tumour, thereby reducing the ratio of activity in tumour to that in normal liver.

**Figure 5** Tumour: normal liver ratio (T:N ratio) of activity before and after the administration of angiotensin II in 48 colorectal hepatic metastases (nine patients).

These considerations would suggest that a normal region should not be selected immediately adjacent to tumour regions when possible. However, if the normal region were at the surface of the liver and the tumour regions deeper within the organ, the effect of attenuation by the surrounding tissue would result in a reduced count density for the deeper tumour regions and the tumour:normal ratio would be decreased.

We defined our normal regions close enough to tumour regions so that differences in attenuation did not significantly effect count densities, but where possible, not so close that

**Figure 4** Computer aligned composite of an albumin colloid slice (green) and corresponding angiotensin II enhanced microsphere slice (red), illustrating a tumour with a hypervascular shell and hypovascular core and accurate tumour therapy.

**Figure 6** The 'improvement' in tumour targeting in 48 colorectal hepatic metastases (nine patients).

$$\text{Improvement} = \frac{\text{Activity in tumour: Activity in liver after angiotensin II}}{\text{Activity in tumour: Activity in liver before angiotensin II}}$$

**Table I** Improvement in tumour uptake of microspheres in nine patients after angiotensin II

| *Patient* | *T:N ratio Before AII (Median)* | *T:N Ratio After AII (Median)* | *Improvement in ratio After AII (Median)* |
|---|---|---|---|
| 1 | 1.60 | 3.36 | 2.98 |
| 2 | 3.96 | 7.62 | 1.92 |
| 3 | 6.04 | 8.10 | 1.11 |
| 4 | 3.73 | 1.54 | 0.52 |
| 5 | 3.40 | 8.82 | 3.41 |
| 6 | 2.57 | 7.61 | 3.03 |
| 7 | 1.33 | 2.64 | 1.77 |
| 8 | 4.65 | 7.32 | 1.74 |
| 9 | 2.44 | 2.61 | 0.86 |

our results were likely to be affected by physiological changes occurring in the normal liver tissue immediately surrounding tumours, or by scatter.

During processing, the images from tumour and normal regions were examined in detail and the effects of the angiotensin II infusion noted. The angiotensin II infusion often appeared to sharpen the tumour outline, thereby slightly reducing the area of the tumour image (compare Figures 2ii and 2iii). One effect of this was to reduce the number of pixels with increased uptake in the angiotensin II study. The corresponding count density (which is calculated using the original number of pixels) was therefore diluted by the empty pixels surrounding the smaller area of increased uptake. This in turn may have led to underestimation of the degree of enhancement produced by angiotensin II.

It is wothy of note that the two patients where angiotensin II enhancement could not be evaluated had exceptionally hypervascular tumours. Whether angiotensin II augmented microsphere concentration within tumours in these patients is largely academic, in view of the efficient delivery of particles to tumour regions under physiological conditions of arterial perfusion.

Any manoeuvre which improves the relative exposure of tumour regions to arterially administered therapeutic agents and reduces hepatotoxicity is likely to improve the regional advantage if the thresholds for drug extraction or metabolism are not exceeded. The temporary nature of the angiotensin II targeting mechanism is ideally suited to bolus injection of microspheres. Targeting would be particularly valuable in a system where a 'sustained release' mechanism of drug administration is in operation, since toxicity in normal tissues may become problematical with continuing and extended drug exposure.

Potential limitations of the use of cytotoxic loaded microspheres in regional hepatic therapy include an increase in arterio-venous shunting within the organ, causing relative loss of regional advantage as the particles enter the systemic-venous circulation and embolise in the pulmonary tissues. However, we have recently shown that base-line shunting in patients with colorectal liver metastases is low and not significantly increased following the regional administration of a 'therapeutic' dose of microspheres (Goldberg *et al.*, 1990*b*). In clinical practice therefore, we have not found arteriovenous shunting to be a problem.

Clearly, this technique will not offer a solution to all patients, since many patients with liver metastases have occult disease elsewhere. However, data from natural history studies suggest that between 20 and 30% of patients with colorectal liver metastases might benefit from hepatic arterial therapy, since this is the proportion of patients in whom disease is thought to be confined to the liver (Welch & Donaldson, 1979; Daly *et al.*, 1985).

In conclusion, we have demonstrated that angiotensin II significantly increases the uptake of microspheres by tumour in patients with colorectal liver metastases. This is likely to enhance the tumour response to cytotoxic loaded microspheres, and reduce hepatotoxicity. We are currently exploring the use of angiotensin II in association with cytotoxic-loaded microspheres in patients with colorectal liver metastases.

We would like to thank The Cancer Research Campaign for their financial assistance.

We are most grateful for the kind assistance of both technical and nursing staff attached to The Department of Nuclear Medicine, Glasgow Royal Infirmary.

We are endebted to Mr Alan Law (Office International, Glasgow) for his assistance with computer equipment.

### References

BERGER, M. (1981). Hepatic infusions for metastatic colorectal carcinoma in a community hospital setting. *Proc. Am. Soc. Clin. Oncol.*, **22**, 456.

BRADPIECE, H.A., BENJAMIN, I.S., HALEVY, A. & BLUMGART, L.H. (1987). Major hepatic resection for colorectal liver metastases. *Br. J. Surg.*, **74**, 324.

DALY, J., BUTLER, J., KEMENY, N. & 5 others (1985). Predicting tumour response in patients with hepatic metastases. *Ann. Surg.*, **202**, 384.

GOLDBERG, J.A., BRADNAM, M.S., KERR, D.J. & 5 others (1987). Arteriovenous shunting of microspheres in patients with colorectal liver metastases: errors in assessment due to free pertechnetate, and the effect of Angiotensin II. *Nucl. Med. Comm.*, **8**, 1033.

GOLDBERG, J.A., MURRAY, T., KERR, D.J. & 4 others (1990*a*). The use of angiotensin II as a potential method of targeting cytotoxic microspheres in patients with intrahepatic tumour. *Br. J. Cancer* (in press).

GOLDBERG, J.A., THOMSON, K., MCCURRACH, G. & 5 others (1990*b*). Arteriovenous shunting in patients with colorectal liver metastases. *Br. J. Cancer* (in press).

KERR, D.J. (1989). 5-Fluorouracil and folinic acid: interesting biochemistry or effective treatment? *Br. J. Cancer*, **60**, 807.

MCARDLE, C.S., LEWI, H., HANSELL, D., KERR, D.J., MCKILLOP, J.H. & WILLMOTT, N. (1988). Cytotoxic-loaded albumin microspheres: a novel approach to regional chemotherapy. *Br. J. Surg.*, **75**, 132.

MALIK, S.T.A. & WRIGLEY, P.F.M. (1988). Intra-arterial hepatic chemotherapy for liver malignancy: not yet proved to prolong survival. *Br. Med. J.*, **297**, 434.

NIELSEN, J., BALSLEV, I. & JENSEN, H.E. (1971). Carcinoma of the colon with liver metastases. Operative indications and prognosis. *Acta Chir. Scand.*, **137**, 463.

O'CONNELL, M.J. (1989). A Phase III trial of 5-fluorouracil and leucovorin in the treatment of advanced colorectal cancer. *Cancer*, **63**, 1026.

RIDGE, J.A., BADING, J.R., GELBARD, A.S., BENUA, R.S. & DALY, J.M. (1987). Perfusion of colorectal hepatic metastases relative distribution of flow from the hepatic artery and portal vein. *Cancer*, **59**, 1547.

SASAKI, Y., IMAOKA, S., HASEGAWA, Y. & 7 others (1985). Changes in distribution of hepatic blood flow induced by intra-arterial infusion of angiotensin II in human hepatic cancer. *Cancer*, **55**, 311.

WADLER, S., SCHWARTZ, E.L., GOLDMAN, M. & 6 others (1989). Fluorouracil and recombinant alfa-2a-interferon: an active regimen against advanced colorectal carcinoma. *J. Clin. Oncol.*, **7**, 1769.

WOOD, C. (1984). Natural history of liver metastases. In: van de Veld, C.J.H. & Sugarbaker, P.H. (eds). *Liver Metastases*, Martinus Nijhoff, pp. 47–54.

WILLMOTT, N., CUMMINGS, J., STUART, J.F.B. & FLORENCE, A.T. (1985). Adriamycin-loaded albumin microspheres: preparation, in vivo distribution and release in the rat. *Biopharmaceutics & Drug Disposition*, **6**, 91.

WELCH, J. & DONALDSON, G.A. (1979). The clinical correlation of an autopsy study of recurrent colorectal cancer. *Ann. Surg.*, **189**, 496.